# Rhus Antidotes

**Published:** 1888-09

**Authors:** James T. Martin


### RHUS ANTIDOTES.

Considerable has been said of late in your journal about the antidotes for Rhus-tox., R. rad. The subject is certainly very interesting, and especially so to one who lives in the vicinity of these plants. Comparing their pathogenesis, they are considered identical, or at least with so little difference that the R. tox. is used where either may be indicated. What real difference there may be is probably due to their habitat. We have on this coast: R. tox. (scarce), R. venenata (not common), R. diversaloba (quite common). Poisoning from this latter variety we are most frequently called upon to antidote. Experience has taught us that your Eastern R. tox. is its best antidote. Then the question comes to us, Why won't our plant (R. diversaloba) be a good antidote for your plant (R. tox.)?

While a plant may antidote itself, it seems to me suicidal to its individuality to do so. If the matter is looked into closely it may be found that a plant best antidotes its cognates.

I am aware that this will open a wide field for discussion, but such things must needs be in order to arrive at the truth. Drugs are supposed to maintain their own individuality, howsoever far they may be attenuated. (If it isn't R. tox. in the cm, then how can you tell what it is?) If this is the case they cannot at any time become antidotal or antagonistic to themselves. The systems of psoric individuals frequently call for certain anti-psoric drugs. So often is this the case that we frequently speak of a Sulphur patient, a Calcarea patient, a Silicea patient, etc.

If any one of these patients should take any crude drug into his system it would produce or bring out the symptoms of the anti-psoric remedy peculiarly adapted to his system, *e. g.*, you doubtless have seen persons take a great deal of crude Sulphur without producing a single Sulphur symptom. He was not a Sulphur patient, and if you will observe closely you may find symptoms of China produced by the irritation of the Sulphur. How often have you seen little children that had taken lime in their milk without ever producing a single Calcarea symptom, while the child was bristling all over with symptoms of some other drug, brought out by the irritation of the crude Calcium. We are willing to admit that if the Sulphur patient takes crude Sulphur into his system, the symptoms produced will be Sulphur and Sulphur will relieve them.

The crude drug in these cases acts as a mechanical irritant, and not as a therapeutic agent that is capable of producing its own individual impression upon the system. In the case of the poisonous plants it is the medicinal irritation instead of the mechanical that we have to deal with, hence a set of symptoms corresponding more nearly to the pathogenesis of the drug taken and the necessity for a direct antidote. This can best be obtained in two ways: first, by a knowledge of the poison met with; second, by an accurate knowledge of its antidotes. A knowledge of the botany of the poisonous plants in your neighborhood will give you a clue to the first, and experience with the antidotal property of your drugs will give you a clue to the second. Further, the plant in the field may be a variety of the same plant of which you have a tincture in your office, in which case you probably possess the means of relieving your patient. In other words, I think that when a doctor is giving R. tox., supposing that he is antidoting R. tox., he is simply mistaken as to the variety of the plant the poison is from which he is trying to neutralize. Hence, I think it necessary for physicians to be conversant with the botany of plants growing in their neighborhoods, and know certainly that they have a case of Rhus tox. poisoning before they report having cured it with that remedy.

JAMES T. MARTIN.

